# An Ishmaelite

**Published:** 1888-09-08

**Authors:** Leslie Keith

**Affiliations:** Author of "The Chilcotes," "Uncle Bob's Niece," "East and West," etc.


### An Ishmaelite.

By Leslie Keith,

Author of "The Chilcotes," "Uncle Bob's Niece," "East and West," etc.

*(Continued from p. 363.)*

### Chapter XXIII.—Vanished!

Arthur was very careful of his fragile companion on the homeward journey. He hurried her away from the Vicarage, where, indeed, they were not pressed to stay.

Cousin Fanny, with a silk handkerchief tied over her head, was dusting, pushing about the disarranged furniture, and clearing the decks after the fight. Nobody, perhaps, but that heroic woman herself knew what a struggle it had been to present so fair a show to the invited guests, and now that the festivities were over the reign of economy was to begin.

She uttered no protest when Arthur told her he had arranged to take Nellie up to town at once.

"I've sent for the fly," he said. "Nellie isn't well; she ought to be at home."

"Of course she ought," said Fanny sharply, "and she ought to stay at home. How can she be well if she's always gadding about—and without her husband too? I call it quite indecent, though I dare say it's the fashion, you will tell me, for young married women to be seen everywhere alone."

Arthur was glad when she quitted reproof to touch on another subject.

"I have written to Cousin Joseph about Clara," she said. "He must have someone to look after the house; it will be the more necessary for him to have a young companion if you are going to leave him. You haven't told me the name of your intended, Arthur."

"I have no right to hope yet," said Arthur, reddening confusedly. "You will hear in good time if I am successful." A sudden gleam of humour flicked over his face as he thought of the shock it would give this good woman when she heard the announcement.

"Well"—Mrs. Coates dismissed Arthur's affairs with chilly indifference—"your uncle can have either Clara or Fanny, but it is Clara's turn, she is the elder."

"And when Clara is disposed of, there will still be Fanny and Minnie and Lettie," thought Arthur to himself. "Uncle Joseph need never lack a housekeeper."

On the backward journey he talked very cheerfully to Nellie; he tried to remember all the pleasant things he had heard of his brother-in-law, and was quite elated when he brought a faint smile to Nellie's pale lips over a little jest that had circulated at the clubs and in which Richard had played quite a brilliant part. To win her to a new interest in her husband, and to bring back her vagrant fancy from its foolish wanderings was not perhaps such a very difficult task. Nellie's weak, diffident nature only needed a little kindness to stimulate her affection and make it cling once more round its legitimate support.

"If only Bellynge will be a little gentle with her," thought Arthur. He persuaded her to go and lie down when they got back to Pont Street, and he put aside his own impatience and waited to see his brother-in-law, who, the servant said, was expected back early. He went into the nursery and played with the boy—a fine, bright little fellow of whom any mother might be proud. He was glad when Nellie's bell rang and she sent for the baby: the little man was her best healer.

When Bellynge came home, Arthur exerted himself to be cordial to this relative whom he did not love.

"I brought Nellie home," he said. "She is lying down; she is not well."

"She seemed well enough yesterday," said Bellynge roughly; "she was well enough to take her own way, and to go to the country, against my wishes."

"Perhaps that has helped on matters," said Arthur, summoning his forbearance. "She looks to me on the edge of a serious illness."

He heard his brother-in-law mount the stair and open the bedroom door and shut it behind him. Then he went away. He could do no more; he could only hope that Bellynge would be patient, and that Nellie, shaken out of her foolish illusion, would turn to him again.

The moment he was free he went off to Bermondsey. He could scarcely curb his impatience long enough to snatch a mouthful of dinner at his club. His absence had given a new force to his determination to win Delia if he could.

He shook himself with a sense of satisfaction when he entered the shabby street. He felt himself at home there; he did not see the bare, sordid side of it all—the abyssmal depths of dulness in which it was plunged; fancy touched it and lifted it into the regions of beauty and delight; turned it into a world of poetry—so strange and all-compelling is the power of love.

The street was unchanged and the shop too. It was the idlest hour of the day when the seekers after food for mind or body were fewest, since it was also the busiest hour in all the surrounding shops and warehouses.

He glanced up over the tall, narrow front, and wondered if Delia was hiding behind a casement and watching his approach: no wispered apprehension came to disturb him; nothing told him that she had been whirled away and safely hidden from him. Fred was there, and Barbara. He grasped his cousin's hand; he took Barbara's with an air of frank and cordial friendship.

"Yes, I've got back," he said. "I came up to-day. The wedding is over, thank goodness—weddings are very funereal affairs, always excepting one's own." He looked at Barbara with a joyous light in his blue eyes.

"Don't ask me any details, Miss Barbara, please. I noticed nothing; I can't even tell you what the bridesmaids wore, though I took one of them in to breakfast. I was thinking of other things. Do you know why I have come to-day? I came to see—no, not you Fred, but your father." He turned again to Barbara. "Can you guess why? Will you wish me success?"

She looked with grave compassion at his bright, frank boyish face.

"Father is in the other shop," she said. "Shall I call him, Mr. Eastgate?"

"No, I will go to him; and afterwards—may I go upstairs?"

She had not the heart to tell him that he would not find the person he sought upstairs. She could not settle to her work when he had hurried away, for wondering what was happening in that interview going on behind the partition. Instinctively she turned to Fred, and their eyes met.

"You knew it!" she said. "It is a pity; you ought to have prevented it."

"I tried to do it," he said. "But I question whether I had any business to interfere. It might be his best happiness I was trying to rob him of."

Barbara shook her head.

"He is very good," she said; "but"—she used the formula he was beginning to hate—"we are different, and it wouldn't be fair to him—or perhaps to Delia either."

In the adjoining shop Mr. Proudie was using the same argument. Miss Betsy's discoveries had perhaps prepared him for Arthur's avowal, and his thoughts had had time to arrange themselves.

"Mr. Arthur, sir," he said, "it's an honour ye are doing me and mine by naming such a thing; but it canna be."

"Why not?"

"My girl's as good a girl as ever stepped, and as bonnie, though I say it that shouldn't; but she's not like the leddies you've been used to, Mr. Arthur, and I couldn't thole to see her looked down upon."

"Looked down upon!" said Arthur, growing nettled. He had made his petition humbly enough. "Do you insinuate such a thing as that I would allow her to be insulted? Who would dare to do it? There is no better lady in the land."

"That's all very well, sir, and I don't misdoubt you; but I've my pride although I'm only a poor tradesman."

"A confounded deal too much pride."

"That's as maybe, Mr. Arthur, but I can't give you my lass. You are an honest and a kind gentleman—there never was a kinder, as I've good cause to know; but there's your folks to think of too, and if you won't think of them, I must. My girl isn't used to the ways of fine ladies and gentlemen—they wouldn't take to her, nor she to them, and maybe—I don't say it wouldn't be nat'rel—maybe a day would come when you would be a bit impatient wi' her, and wish that you had chose different."

"Proudie," said Arthur with some hautiness, "you do me great injustice. Have I ever proposed to separate your child from you, or to take her into a world where she would not feel at ease?"

"It would be done, all the same, Mr. Arthur," said the sagacious Scotchman. "You're a gentleman, you can't change that."

"I hope I shall always be a gentleman," said Arthur fiercely. "I hope I am enough of one to shield my wife from so much as a hint of discomfort. I will cast in my lot with her; I will come here and live beside you; you shall not be separated from her."

"No, no; that would be offering up the man instead of the woman," said Proudie with a smile. "Folks should marry in their own degree, Mr. Arthur; there's no happiness unless."

"Proudie," said Arthur, 'I can't take my dismissal from you; I come to you in all good faith, because I love your daughter, and will count it the best good of my life to win her. I have nobody to whom to account for my actions. I am free; I have money enough to live where I like and to do as I like, and I have a right to choose as I will. I can't take No from you; you have nothing to urge against me except my position, and I have shown you that I can relinquish that, if you ask it of me, without so much as a sigh. I refuse to take my dismissal, except from Delia. If she bids me go, I will try to bear it, and will see her no more; but I must see her."

"She isn't here, Mr. Arthur, she's away."

"You have sent her away!" cried Arthur, his anger flaming out.

"We thought it best—her mother and me," said Proudie firmly. "We thought of the lassie first, maybe, being our own; but we had you in our minds too, Mr. Arthur."

"Thank you! You did me a great service, truly."

"The day may come when you'll think it," said Proudie with unruffled compose; "and if ever it should come, Mr. Arthur, I'll be the first to agree with you; and I thank you kindly for wishing so well by us."

"Do you suppose you have done with me?" asked Arthur savagely. "Haven't I said that I would take my answer from on one but Delia? You have sent her away, but I will find her, wherever she is. My love would be as poor an affair as you seem to think it, if it could yield to the first argument."

He was wrathful, hurt, and disappointed, but neither his menaces nor his entreaties moved the father: the Scotchman's native pride sustained him. He was firm as a rock in refusing to surrender Delia's address. No man or woman should have it in his power to say that he had angled for the prize and secured it. He would rather break Delia's heart than that.

It was only towards the end of a long and stormy interview that he gave a reluctant consent to Arthur's reopening the question in the future. If he remained in the same mind in six months' time, he might then once more approach the subject. Probably Proudie had faith in the curative value of six months' absence from the beloved object. Absence is said to make the heart grow fonder, it is true; but there is an adage of equal value which asserts that what the eye does not see the heart does not grieve for. A good many bereavements are comfortably got over in a lesser space of time than was allotted to this fond lover.

"But mind, I promise nothing," cried the cautious Scot.

Arthur gave a laugh half scornful, half sad.

"I will win her in spite of you, Proudie," he said. "Six months! I would serve her for six years."

*(To be continued.)*

